
JOURNAL INFORMATION
==============================
NLM Title Abbreviation: Can Med Educ J
Journal ID: Can Med Educ J
NLM Journal ID: 101560935
Journal ID: CMEJ

Canadian Medical Education Journal

EISSN: 1923-1202
Publisher: University of Saskatchewan

ARTICLE INFORMATION
==============================
PMCID: PMC6448505
Article ID: CMEJ-10-e198
Article version: 1
Subjects: Abstracts

Dedicated Poster Abstracts

Electronic publication date: 2019 Apr 3
Publication date: 2019 Apr
Volume: 10
Issue: 2
First page: e198
Last page: e256
Copyright year: 2019
License: This is an Open Journal Systems article distributed under the terms of the Creative Commons Attribution License (http://creativecommons.org/licenses/by/2.0) which permits unrestricted use, distribution, and reproduction in any medium, provided the original work is properly cited.
License URL: https://creativecommons.org/licenses/by/2.0/

==============================
JOURNAL INFORMATION
==============================
NLM Title Abbreviation: Can Med Educ J
Journal ID: Can Med Educ J
NLM Journal ID: 101560935
Journal ID: CMEJ

Canadian Medical Education Journal

EISSN: 1923-1202
Publisher: University of Saskatchewan

ARTICLE INFORMATION
==============================
Subjects: Dp 1–3

CaRMS Portfolio Evaluation: Are there any pearls in the oysters?

Dore Kelly McMaster University

Fedorkow Donna McMaster University

Lamont John McMaster University

Azzam Joseph McMaster University

Dzaja Nancy McMaster University

Electronic publication date: 2019 Apr 3

JOURNAL INFORMATION
==============================
NLM Title Abbreviation: Can Med Educ J
Journal ID: Can Med Educ J
NLM Journal ID: 101560935
Journal ID: CMEJ

Canadian Medical Education Journal

EISSN: 1923-1202
Publisher: University of Saskatchewan

ARTICLE INFORMATION
==============================
Subjects: Dp 1–4

National Resident Selection Practices

Schabort Inge McMaster University

Dore Kelly McMaster University

Bandiera Glen University of Toronto

Cervin Cathy Northern Ontario School of Medicine

Russell Dana McMaster University

Kreuger Sharyn McMaster University

Electronic publication date: 2019 Apr 3

JOURNAL INFORMATION
==============================
NLM Title Abbreviation: Can Med Educ J
Journal ID: Can Med Educ J
NLM Journal ID: 101560935
Journal ID: CMEJ

Canadian Medical Education Journal

EISSN: 1923-1202
Publisher: University of Saskatchewan

ARTICLE INFORMATION
==============================
Subjects: Dp 1–5

Defining Applicant Attributes and a Brand for Child and Adolescent Psychiatry (CAP) Subspecialty Selection at University of Toronto (U of T)

Kulkarni Chetana University of Toronto

D. Hanson Mark University of Toronto

Rasasingham Raj University of Toronto

Gorman Daniel University of Toronto

Woods Nikki University of Toronto

Kulasegaram Kulamaken (Mahan) University of Toronto

Szatmari Peter University of Toronto,

Electronic publication date: 2019 Apr 3

JOURNAL INFORMATION
==============================
NLM Title Abbreviation: Can Med Educ J
Journal ID: Can Med Educ J
NLM Journal ID: 101560935
Journal ID: CMEJ

Canadian Medical Education Journal

EISSN: 1923-1202
Publisher: University of Saskatchewan

ARTICLE INFORMATION
==============================
Subjects: Dp 1–7

Beyond GPAs, MCAT scores, MMIs, and aspects of randomness: In pursuit of increased equity in the medical school admissions process at the University of Calgary

Doig Christopher University of Calgary

Myhre Doug University of Calgary

Walker Ian University of Calgary

Greer Gretchen University of Calgary

Electronic publication date: 2019 Apr 3

JOURNAL INFORMATION
==============================
NLM Title Abbreviation: Can Med Educ J
Journal ID: Can Med Educ J
NLM Journal ID: 101560935
Journal ID: CMEJ

Canadian Medical Education Journal

EISSN: 1923-1202
Publisher: University of Saskatchewan

ARTICLE INFORMATION
==============================
Subjects: Dp 1–8

Carrying the Weight of Lower Childhood Socioeconomic Status into Medical Schoo

Lam Justin University of Toronto

Babcock Glenys University of Toronto

Ruetalo Mariela University of Toronto

Latter David University of Toronto

Okafor Ike University of Toronto

Balakrishna Anita University of Toronto

Robinson Lisa University of Toronto

Electronic publication date: 2019 Apr 3

JOURNAL INFORMATION
==============================
NLM Title Abbreviation: Can Med Educ J
Journal ID: Can Med Educ J
NLM Journal ID: 101560935
Journal ID: CMEJ

Canadian Medical Education Journal

EISSN: 1923-1202
Publisher: University of Saskatchewan

ARTICLE INFORMATION
==============================
Subjects: Dp 2–2

An Innovative Course on Leadership to Enhance Students' Ability to Influence Practice Change

Gerber Patricia University of British Columbia

Electronic publication date: 2019 Apr 3

JOURNAL INFORMATION
==============================
NLM Title Abbreviation: Can Med Educ J
Journal ID: Can Med Educ J
NLM Journal ID: 101560935
Journal ID: CMEJ

Canadian Medical Education Journal

EISSN: 1923-1202
Publisher: University of Saskatchewan

ARTICLE INFORMATION
==============================
Subjects: Dp 2–3

Transformative Learning: Global Health Observerships improve Cultural Sensitivity and Personal Advocacy

Soleas Eleftherios Queen’s University

Matzinger Elizabeth Queen’s University

Sheahan Guy Queen’s University

Chan Linda Queen’s University

Carpenter Jennifer Queen’s University

De sousa Mikaila Queen’s University

Baumhour Jessica Queen’s University

Electronic publication date: 2019 Apr 3

JOURNAL INFORMATION
==============================
NLM Title Abbreviation: Can Med Educ J
Journal ID: Can Med Educ J
NLM Journal ID: 101560935
Journal ID: CMEJ

Canadian Medical Education Journal

EISSN: 1923-1202
Publisher: University of Saskatchewan

ARTICLE INFORMATION
==============================
Subjects: Dp 2–4

How Learners in Diverse Contexts Conceptualize Health Advocacy: Implications for Practice and Education

Hubinette Maria University of British Columbia

Kahlke Renate The Royal College of Physicians and Surgeons

van der Goes Theresa University of British Columbia

Clark Jenn University of British Columbia

Scott Ian University of British Columbia

Electronic publication date: 2019 Apr 3

JOURNAL INFORMATION
==============================
NLM Title Abbreviation: Can Med Educ J
Journal ID: Can Med Educ J
NLM Journal ID: 101560935
Journal ID: CMEJ

Canadian Medical Education Journal

EISSN: 1923-1202
Publisher: University of Saskatchewan

ARTICLE INFORMATION
==============================
Subjects: Dp 2–5

Innovating with Advocacy: Evaluation of a Novel Advocacy Project in the Family Medicine Block Clerkship Curriculum

Owen James University of Toronto

Maker Dara University of Toronto

Nyhof-Young Joyce University of Toronto

Electronic publication date: 2019 Apr 3

JOURNAL INFORMATION
==============================
NLM Title Abbreviation: Can Med Educ J
Journal ID: Can Med Educ J
NLM Journal ID: 101560935
Journal ID: CMEJ

Canadian Medical Education Journal

EISSN: 1923-1202
Publisher: University of Saskatchewan

ARTICLE INFORMATION
==============================
Subjects: Dp 2–7

Leadership Education for Advancing Practice Simulation Course (LEAP-S): Design and evaluation methodology

Thakur Anupam Centre for Addiction and Mental Health, Toronto

Meschino Diane University of Toronto

Nirula Latika Centre for Addiction and Mental Health

Naismith Laura Centre for Addiction and Mental Health

Gordon Tucker Centre for Addiction and Mental Health

Saiva Anika Centre for Addiction and Mental Health

Electronic publication date: 2019 Apr 3

JOURNAL INFORMATION
==============================
NLM Title Abbreviation: Can Med Educ J
Journal ID: Can Med Educ J
NLM Journal ID: 101560935
Journal ID: CMEJ

Canadian Medical Education Journal

EISSN: 1923-1202
Publisher: University of Saskatchewan

ARTICLE INFORMATION
==============================
Subjects: Dp 2–8

Teaching Leadership in Medical School: A Challenging, yet Crucial Task

Doshi Samik University of Toronto

Pham Thuy-Nga University of Toronto

Electronic publication date: 2019 Apr 3

JOURNAL INFORMATION
==============================
NLM Title Abbreviation: Can Med Educ J
Journal ID: Can Med Educ J
NLM Journal ID: 101560935
Journal ID: CMEJ

Canadian Medical Education Journal

EISSN: 1923-1202
Publisher: University of Saskatchewan

ARTICLE INFORMATION
==============================
Subjects: Dp 3–2

Educating for Lifelong Learning: Rendering explicit strategies/activities used in health science programs

Xhignesse Marianne Université de Sherbrooke

Mathieu Luc Université de Sherbrooke

Clavet Diane Université de Sherbrooke

Electronic publication date: 2019 Apr 3

JOURNAL INFORMATION
==============================
NLM Title Abbreviation: Can Med Educ J
Journal ID: Can Med Educ J
NLM Journal ID: 101560935
Journal ID: CMEJ

Canadian Medical Education Journal

EISSN: 1923-1202
Publisher: University of Saskatchewan

ARTICLE INFORMATION
==============================
Subjects: Dp 3–6

Developing a Framework of Integrated Competencies for Adaptive Expertise in Integrated Physical and Mental Health Care

Sockalingam Sanjeev University of Toronto

Chaudhary Zarah University of Toronto

Barnett Rachael University of Toronto

Lazor Jana University of Toronto

Mylopoulos Maria University of Toronto,

Electronic publication date: 2019 Apr 3

JOURNAL INFORMATION
==============================
NLM Title Abbreviation: Can Med Educ J
Journal ID: Can Med Educ J
NLM Journal ID: 101560935
Journal ID: CMEJ

Canadian Medical Education Journal

EISSN: 1923-1202
Publisher: University of Saskatchewan

ARTICLE INFORMATION
==============================
Subjects: Dp 3–7

The Making of an MFM Specialist: How defining competency can improve fellowship training in Maternal Fetal Medicine

Williamson Monica University of Toronto

Czikk Marie University of Toronto

Rojas Gualdron David University of Toronto

Electronic publication date: 2019 Apr 3

JOURNAL INFORMATION
==============================
NLM Title Abbreviation: Can Med Educ J
Journal ID: Can Med Educ J
NLM Journal ID: 101560935
Journal ID: CMEJ

Canadian Medical Education Journal

EISSN: 1923-1202
Publisher: University of Saskatchewan

ARTICLE INFORMATION
==============================
Subjects: Dp 3–8

Research Productivity in Physical Medicine & Rehabilitation: Are Publications an Appropriate Metric for Evaluating Research Participation in CBME?

Ali Bateman E. Western University

Teasell Robert Western University

Electronic publication date: 2019 Apr 3

JOURNAL INFORMATION
==============================
NLM Title Abbreviation: Can Med Educ J
Journal ID: Can Med Educ J
NLM Journal ID: 101560935
Journal ID: CMEJ

Canadian Medical Education Journal

EISSN: 1923-1202
Publisher: University of Saskatchewan

ARTICLE INFORMATION
==============================
Subjects: Dp 4–1

Complex Intrinsic Skill Competencies: A Fit-for-Purpose Multiple Component Assessment Tool

Sibbald Debra Touchstone Institute

Monteiro Sandra Touchstone Institute

Electronic publication date: 2019 Apr 3

JOURNAL INFORMATION
==============================
NLM Title Abbreviation: Can Med Educ J
Journal ID: Can Med Educ J
NLM Journal ID: 101560935
Journal ID: CMEJ

Canadian Medical Education Journal

EISSN: 1923-1202
Publisher: University of Saskatchewan

ARTICLE INFORMATION
==============================
Subjects: Dp 4–5

Using Borderline Regression Method (BRM) as Standard Setting in Objective Structured Clinical Examinations (OSCEs): Empirical findings from MD Program, University of Toronto

Tu Yuxin University of Toronto

Charles Tamica University of Toronto

Pittini Richard University of Toronto

Tait Glendon University of Toronto

(Mahan) Kulasegaram Kulamakan University of Toronto

Tzanetos Katina University of Toronto

Howard Frazer University of Toronto

Electronic publication date: 2019 Apr 3

JOURNAL INFORMATION
==============================
NLM Title Abbreviation: Can Med Educ J
Journal ID: Can Med Educ J
NLM Journal ID: 101560935
Journal ID: CMEJ

Canadian Medical Education Journal

EISSN: 1923-1202
Publisher: University of Saskatchewan

ARTICLE INFORMATION
==============================
Subjects: Dp 5–1

Factors influencing family physicians referral for bariatric surgery: A systematic review

Cofie Nicholas Queen’s University

Sivapalan Nardhana Queen’s University

Chan Linda McMaster University

Barber David Queen’s University

Dalgarno Nancy Queen’s University

Zevin Boris Queen’s University

Electronic publication date: 2019 Apr 3

JOURNAL INFORMATION
==============================
NLM Title Abbreviation: Can Med Educ J
Journal ID: Can Med Educ J
NLM Journal ID: 101560935
Journal ID: CMEJ

Canadian Medical Education Journal

EISSN: 1923-1202
Publisher: University of Saskatchewan

ARTICLE INFORMATION
==============================
Subjects: Dp 5–2

Integrating Continuing Professional Development to Support Team-based Primary Care

Newton Christie University of British Columbia

Wood Victoria University of British Columbia

Lynn Brenna University of British Columbia

Bluman Bob University of British Columbia

Electronic publication date: 2019 Apr 3

JOURNAL INFORMATION
==============================
NLM Title Abbreviation: Can Med Educ J
Journal ID: Can Med Educ J
NLM Journal ID: 101560935
Journal ID: CMEJ

Canadian Medical Education Journal

EISSN: 1923-1202
Publisher: University of Saskatchewan

ARTICLE INFORMATION
==============================
Subjects: Dp 5–3

Valuing Perspectives: streamlining CFPC and RCPSC accreditation requirements in a single user-friendly online application

Cuddy Trevor University of Toronto

Schneeweiss Suzan University of Toronto

Paton Morag University of Toronto

Jones Renice University of Toronto

Farah Karma University of Toronto

McConnell Katherine University of Toronto

Kuepers Carlo University of Toronto

Tipping Jane University of Toronto

Electronic publication date: 2019 Apr 3

JOURNAL INFORMATION
==============================
NLM Title Abbreviation: Can Med Educ J
Journal ID: Can Med Educ J
NLM Journal ID: 101560935
Journal ID: CMEJ

Canadian Medical Education Journal

EISSN: 1923-1202
Publisher: University of Saskatchewan

ARTICLE INFORMATION
==============================
Subjects: Dp 5–4

Pigs' Feet and Oranges as Simulation Tools: Small Group Practical Learning of Basic Dermatological Procedures for Primary Care Providers

Asai Yuka Queen’s University

Evans Katie Queen’s University

McDiarmid Laura Queen’s University

Soleas Eleftherios Queen’s University

Mangan Cynthia Queen’s University

Smith Karen Queen’s University

Electronic publication date: 2019 Apr 3

JOURNAL INFORMATION
==============================
NLM Title Abbreviation: Can Med Educ J
Journal ID: Can Med Educ J
NLM Journal ID: 101560935
Journal ID: CMEJ

Canadian Medical Education Journal

EISSN: 1923-1202
Publisher: University of Saskatchewan

ARTICLE INFORMATION
==============================
Subjects: Dp 5–5

Flipping a CPD class for active learning: Lessons learned from repackaging an ECG interpretation course for physicians

Weeks Sarah University of Calgary

Burnett Chloe University of Calgary

Dongo Tendai University of Calgary

Ryan Caitlin University of Calgary

McMeekin James University of Calgary

Quinn Russell University of Calgary

Gorsche Ronald University of Calgary

Burak Kelly University of Calgary

Electronic publication date: 2019 Apr 3

JOURNAL INFORMATION
==============================
NLM Title Abbreviation: Can Med Educ J
Journal ID: Can Med Educ J
NLM Journal ID: 101560935
Journal ID: CMEJ

Canadian Medical Education Journal

EISSN: 1923-1202
Publisher: University of Saskatchewan

ARTICLE INFORMATION
==============================
Subjects: Dp 5–6

Building Competencies for CPD Providers: An innovative approach to CPD faculty development

Schneeweiss Suzan University of Toronto

Tipping Jane University of Toronto

Electronic publication date: 2019 Apr 3

JOURNAL INFORMATION
==============================
NLM Title Abbreviation: Can Med Educ J
Journal ID: Can Med Educ J
NLM Journal ID: 101560935
Journal ID: CMEJ

Canadian Medical Education Journal

EISSN: 1923-1202
Publisher: University of Saskatchewan

ARTICLE INFORMATION
==============================
Subjects: Dp 5–8

A Pan-Canadian Survey of CPD Research Activity

MacLeod Tanya Dalhousie University

Luconi Francesca McGill

Paton Morag University of Toronto

Teferra Meron McGill

Wooster Elizabeth University of Toronto

Electronic publication date: 2019 Apr 3

JOURNAL INFORMATION
==============================
NLM Title Abbreviation: Can Med Educ J
Journal ID: Can Med Educ J
NLM Journal ID: 101560935
Journal ID: CMEJ

Canadian Medical Education Journal

EISSN: 1923-1202
Publisher: University of Saskatchewan

ARTICLE INFORMATION
==============================
Subjects: Dp 6–1

An Evaluation of Geriatric Psychiatry Education in Canada

Andrew Melissa Queen’s University

Hussain Maria Queen’s University

Zarzour Colin University of Ottawa

Andrew Melissa Queen’s University

Braund Heather Queen’s University

Dalgarno Nancy Queen’s University

Egan Rylan Queen’s University

Electronic publication date: 2019 Apr 3

JOURNAL INFORMATION
==============================
NLM Title Abbreviation: Can Med Educ J
Journal ID: Can Med Educ J
NLM Journal ID: 101560935
Journal ID: CMEJ

Canadian Medical Education Journal

EISSN: 1923-1202
Publisher: University of Saskatchewan

ARTICLE INFORMATION
==============================
Subjects: Dp 6–5

A framework for incorporating discussion of social identities into Case-Based Learning

Balbaa Amira University of Toronto

Balbaa* Amira University of Toronto

Berditchevskaia* Inna University of Toronto

Bowden* Sylvie University of Toronto

Freeman* Sarah University of Toronto

Kirubarajan* Abirami University of Toronto

Klostermann* Natalie University of Toronto

Naguib* Mariam University of Toronto

Lazor Jana University of Toronto

Electronic publication date: 2019 Apr 3

JOURNAL INFORMATION
==============================
NLM Title Abbreviation: Can Med Educ J
Journal ID: Can Med Educ J
NLM Journal ID: 101560935
Journal ID: CMEJ

Canadian Medical Education Journal

EISSN: 1923-1202
Publisher: University of Saskatchewan

ARTICLE INFORMATION
==============================
Subjects: Dp 6–6

ReCAPTCHA my Curriculum: Internet-inspired methodology to identify curricular gaps using student performance with off the shelf examinations.

Pinsk Maury University of Manitoba

Cohen Barry University of Manitoba

Ripstein Ira University of Manitoba

Hyman Jeff University of Manitoba

Postalow Fabiana University of Manitoba

Li William University of Manitoba

Ballinger Karen University of Manitoba

Lautatzis Maria-Elena University of Manitoba

Wosnitza Kari University of Manitoba

Electronic publication date: 2019 Apr 3

JOURNAL INFORMATION
==============================
NLM Title Abbreviation: Can Med Educ J
Journal ID: Can Med Educ J
NLM Journal ID: 101560935
Journal ID: CMEJ

Canadian Medical Education Journal

EISSN: 1923-1202
Publisher: University of Saskatchewan

ARTICLE INFORMATION
==============================
Subjects: Dp 7–2

Stigma in medical education: student experiences and insights

Riou Kylie University of Saskatchewan

Bennett Vern University of Saskatchewan

Teucher Ulrich University of Saskatchewan

D'Eon Marcel University of Saskatchewan

Electronic publication date: 2019 Apr 3

JOURNAL INFORMATION
==============================
NLM Title Abbreviation: Can Med Educ J
Journal ID: Can Med Educ J
NLM Journal ID: 101560935
Journal ID: CMEJ

Canadian Medical Education Journal

EISSN: 1923-1202
Publisher: University of Saskatchewan

ARTICLE INFORMATION
==============================
Subjects: Dp 7–3

Student outcomes of service learning activity within undergraduate medical curriculum

Nanna Urarat Department of Preclinical Science, Faculty of Medicine, Thammasat University

Chularojanamontri Linda Department of Preclinical Science, Faculty of Medicine, Thammasat University

Chaikan Ammara Department of Preclinical Science, Faculty of Medicine, Thammasat University

Malisorn Nipapan Department of Preclinical Science, Faculty of Medicine, Thammasat University

Electronic publication date: 2019 Apr 3

JOURNAL INFORMATION
==============================
NLM Title Abbreviation: Can Med Educ J
Journal ID: Can Med Educ J
NLM Journal ID: 101560935
Journal ID: CMEJ

Canadian Medical Education Journal

EISSN: 1923-1202
Publisher: University of Saskatchewan

ARTICLE INFORMATION
==============================
Subjects: Dp 7–4

"Equity and Diversity in CME and CPD: demographic assessment as a tool to identify disparities."

Plens Shecaira Laura McMaster University

Azzam Khalid McMaster University

Silla Angela McMaster University

Electronic publication date: 2019 Apr 3

JOURNAL INFORMATION
==============================
NLM Title Abbreviation: Can Med Educ J
Journal ID: Can Med Educ J
NLM Journal ID: 101560935
Journal ID: CMEJ

Canadian Medical Education Journal

EISSN: 1923-1202
Publisher: University of Saskatchewan

ARTICLE INFORMATION
==============================
Subjects: Dp 7–5

Fit In, Don't Fit in: An Exploration of What MD Students Are Feeling

Ruetalo Mariela University of Toronto

Babcock Glenys University of Toronto

Robinson Lisa University of Toronto

Electronic publication date: 2019 Apr 3

JOURNAL INFORMATION
==============================
NLM Title Abbreviation: Can Med Educ J
Journal ID: Can Med Educ J
NLM Journal ID: 101560935
Journal ID: CMEJ

Canadian Medical Education Journal

EISSN: 1923-1202
Publisher: University of Saskatchewan

ARTICLE INFORMATION
==============================
Subjects: Dp 7–6

An Appreciative Inquiry to Social Accountability in Canada

Walling Erin University of Saskatchewan

Woollard Robert University of British Columbia

Wasik Adrienne Research Consultant

Woollard Robert University of British Columbia

Lachance Eric Université de Sherbrooke

Electronic publication date: 2019 Apr 3

JOURNAL INFORMATION
==============================
NLM Title Abbreviation: Can Med Educ J
Journal ID: Can Med Educ J
NLM Journal ID: 101560935
Journal ID: CMEJ

Canadian Medical Education Journal

EISSN: 1923-1202
Publisher: University of Saskatchewan

ARTICLE INFORMATION
==============================
Subjects: Dp 8–3

Competence in Context: Building InSIGHTful Global Health Learning Opportunities

Allison Jill Memorial – University of Newfoundland

Mulay Shree Memorial – University of Newfoundland

Electronic publication date: 2019 Apr 3

JOURNAL INFORMATION
==============================
NLM Title Abbreviation: Can Med Educ J
Journal ID: Can Med Educ J
NLM Journal ID: 101560935
Journal ID: CMEJ

Canadian Medical Education Journal

EISSN: 1923-1202
Publisher: University of Saskatchewan

ARTICLE INFORMATION
==============================
Subjects: Dp 8–5

Identifying strengths and challenges within surgical programs: An exploration of learning environment and learning culture

Saxena Anurag University of Saskatchewan

Desanghere Loni University of Saskatchewan

Robertson-Frey Tanya University of Saskatchewan

Lawrence Kathy University of Saskatchewan

Hayes Paul University of Saskatchewan

Thiel John University of Saskatchewan

Mendez Ivar University of Saskatchewan

Electronic publication date: 2019 Apr 3

JOURNAL INFORMATION
==============================
NLM Title Abbreviation: Can Med Educ J
Journal ID: Can Med Educ J
NLM Journal ID: 101560935
Journal ID: CMEJ

Canadian Medical Education Journal

EISSN: 1923-1202
Publisher: University of Saskatchewan

ARTICLE INFORMATION
==============================
Subjects: Dp 8–6

Campus décentralisé de l'Université de Montréal en Mauricie : Mission sociale accomplie après les 10 premières cohortes diplômées

Girouard Marie-Hélène Université de Montréal

Electronic publication date: 2019 Apr 3

JOURNAL INFORMATION
==============================
NLM Title Abbreviation: Can Med Educ J
Journal ID: Can Med Educ J
NLM Journal ID: 101560935
Journal ID: CMEJ

Canadian Medical Education Journal

EISSN: 1923-1202
Publisher: University of Saskatchewan

ARTICLE INFORMATION
==============================
Subjects: Dp 8–8

Empathy erosion in medical training: A qualitative study of trainee perspectives

Zhou Linghong University of British Columbia

Riviere Raphael University of Ottawa

Byszewski Anna University of Ottawa

Lochnan Heather University of Ottawa

Electronic publication date: 2019 Apr 3

JOURNAL INFORMATION
==============================
NLM Title Abbreviation: Can Med Educ J
Journal ID: Can Med Educ J
NLM Journal ID: 101560935
Journal ID: CMEJ

Canadian Medical Education Journal

EISSN: 1923-1202
Publisher: University of Saskatchewan

ARTICLE INFORMATION
==============================
Subjects: Dp 9–1

Apprentissage des habiletés d'entrevue : former des patients simulés formateurs comme tuteur d'apprentissage

Burnier Isabelle University of Ottawa

Bouchard-Lamothe Diane University of Ottawa

Tremblay Manon chercheuse indépendante

Electronic publication date: 2019 Apr 3

JOURNAL INFORMATION
==============================
NLM Title Abbreviation: Can Med Educ J
Journal ID: Can Med Educ J
NLM Journal ID: 101560935
Journal ID: CMEJ

Canadian Medical Education Journal

EISSN: 1923-1202
Publisher: University of Saskatchewan

ARTICLE INFORMATION
==============================
Subjects: Dp 9–5

Mapping key competencies in sustainability : a case study of Université Laval's doctoral degree in medicine

Thériault Julie Université Laval

Forget Daniel Université Laval

Lamarre Martin Université Laval

Lemieux Roxanne Université Laval

Bujold Gabrielle Université Laval

Electronic publication date: 2019 Apr 3

JOURNAL INFORMATION
==============================
NLM Title Abbreviation: Can Med Educ J
Journal ID: Can Med Educ J
NLM Journal ID: 101560935
Journal ID: CMEJ

Canadian Medical Education Journal

EISSN: 1923-1202
Publisher: University of Saskatchewan

ARTICLE INFORMATION
==============================
Subjects: Dp 9–6

Une démarche de conception d'activités d'enseignement en partenariat étroit avec des patients sur leur expérience vécue

Boire-Lavigne Anne-Marie Université de Sherbrooke

Laberge Stéphane Université de Sherbrooke

Bernard Jocelyn Université de Sherbrooke

Dumas Marc Université de Sherbrooke

Garde-Granger Perrine Université de Sherbrooke

Héon-Lepage Michèle Université de Sherbrooke

Lafrenaye Sylvie Université de Sherbrooke

Le Sieur Iris Université de Sherbrooke

Drouin Guy Université de Sherbrooke

Clavet Diane Université de Sherbrooke

Electronic publication date: 2019 Apr 3

JOURNAL INFORMATION
==============================
NLM Title Abbreviation: Can Med Educ J
Journal ID: Can Med Educ J
NLM Journal ID: 101560935
Journal ID: CMEJ

Canadian Medical Education Journal

EISSN: 1923-1202
Publisher: University of Saskatchewan

ARTICLE INFORMATION
==============================
Subjects: Dp 9–8

The Face of Oasis: Understanding Youth Mental Health Issues through Portraiture and Narrative

Stewart Wendy Dalhousie University

Gilbert Mark Dalhousie University

Electronic publication date: 2019 Apr 3

JOURNAL INFORMATION
==============================
NLM Title Abbreviation: Can Med Educ J
Journal ID: Can Med Educ J
NLM Journal ID: 101560935
Journal ID: CMEJ

Canadian Medical Education Journal

EISSN: 1923-1202
Publisher: University of Saskatchewan

ARTICLE INFORMATION
==============================
Subjects: Dp 10–2

Medical Assistance in Dying: A Resource Library for Practitioners

Coderre-Ball Angela Queen’s University

Dalgarno Nancy Queen’s University

Braund Heather Queen’s University

Soleas Eleftherios Queen’s University

Fraser Bryn Queen’s University

Egan Rylan Queen’s University

Electronic publication date: 2019 Apr 3

JOURNAL INFORMATION
==============================
NLM Title Abbreviation: Can Med Educ J
Journal ID: Can Med Educ J
NLM Journal ID: 101560935
Journal ID: CMEJ

Canadian Medical Education Journal

EISSN: 1923-1202
Publisher: University of Saskatchewan

ARTICLE INFORMATION
==============================
Subjects: Dp 10–5

Development of Nationally-Validated Competencies for Medical Undergraduates in Palliative and End-of-Life Care

Boyle Anne McMaster University

H. Bush Shirley University of Ottawa

Chary Srini University of Calgary

Roze des Ordons Amanda University of Calgary

Electronic publication date: 2019 Apr 3

JOURNAL INFORMATION
==============================
NLM Title Abbreviation: Can Med Educ J
Journal ID: Can Med Educ J
NLM Journal ID: 101560935
Journal ID: CMEJ

Canadian Medical Education Journal

EISSN: 1923-1202
Publisher: University of Saskatchewan

ARTICLE INFORMATION
==============================
Subjects: Dp 10–6

Dynamically using rotation capacity to optimize our Clerkship Lottery scheduling method

Paget Mike University of Calgary

Liu Chaoji University of Calgary

Veale Pamela University of Calgary

Electronic publication date: 2019 Apr 3

JOURNAL INFORMATION
==============================
NLM Title Abbreviation: Can Med Educ J
Journal ID: Can Med Educ J
NLM Journal ID: 101560935
Journal ID: CMEJ

Canadian Medical Education Journal

EISSN: 1923-1202
Publisher: University of Saskatchewan

ARTICLE INFORMATION
==============================
Subjects: Dp 11–1

Supporting and Training Moderators of Online CPD Courses

Hazelton Lara Dalhousie University

Love Susan Dalhousie University

Bonang Lisa Dalhousie University

Electronic publication date: 2019 Apr 3

JOURNAL INFORMATION
==============================
NLM Title Abbreviation: Can Med Educ J
Journal ID: Can Med Educ J
NLM Journal ID: 101560935
Journal ID: CMEJ

Canadian Medical Education Journal

EISSN: 1923-1202
Publisher: University of Saskatchewan

ARTICLE INFORMATION
==============================
Subjects: Dp 11–2

MacAdemia: a novel faculty development program that meets the needs of distributed medical educators at McMaster University

Catherine Tong X. McMaster University

Kundi Anjali McMaster University

Bell Amanda McMaster University

Morris Cathy McMaster University

Wong Anne McMaster University

Electronic publication date: 2019 Apr 3

JOURNAL INFORMATION
==============================
NLM Title Abbreviation: Can Med Educ J
Journal ID: Can Med Educ J
NLM Journal ID: 101560935
Journal ID: CMEJ

Canadian Medical Education Journal

EISSN: 1923-1202
Publisher: University of Saskatchewan

ARTICLE INFORMATION
==============================
Subjects: Dp 11–4

Edu-cafes - an opportunity for faculty development?

McClennan Sarah University of Toronto

Lazor Jana University of Toronto

Electronic publication date: 2019 Apr 3

JOURNAL INFORMATION
==============================
NLM Title Abbreviation: Can Med Educ J
Journal ID: Can Med Educ J
NLM Journal ID: 101560935
Journal ID: CMEJ

Canadian Medical Education Journal

EISSN: 1923-1202
Publisher: University of Saskatchewan

ARTICLE INFORMATION
==============================
Subjects: Dp 11–5

Better Together: A new model for supporting education scholarship

Nyhof-Young Joyce University of Toronto

Onyura Betty University of Toronto

Bordman Risa University of Ottawa

Ellis Rachel University of Toronto

Nutik Melissa University of Toronto

Woods Nicole University of Toronto

Wright Sarah University of Toronto

Freeman Risa University of Toronto

Forte Milena University of Toronto

Kulasegaram Mahan University of Toronto

Electronic publication date: 2019 Apr 3

JOURNAL INFORMATION
==============================
NLM Title Abbreviation: Can Med Educ J
Journal ID: Can Med Educ J
NLM Journal ID: 101560935
Journal ID: CMEJ

Canadian Medical Education Journal

EISSN: 1923-1202
Publisher: University of Saskatchewan

ARTICLE INFORMATION
==============================
Subjects: Dp 11–6

What is the Resident Perspective on Quality Improvement Education in Family Medicine Residency Programs in Canada?

Cole Kelsi McMaster University

Cole Kelsi McMaster University

Tissera Hiromi McGill

Hortas-Laberge Camille Université Laval

Chhina Jason University of British Columbia

Chan Derek University of Alberta

Krol-Kennedy Alicja University of Calgary

Electronic publication date: 2019 Apr 3

JOURNAL INFORMATION
==============================
NLM Title Abbreviation: Can Med Educ J
Journal ID: Can Med Educ J
NLM Journal ID: 101560935
Journal ID: CMEJ

Canadian Medical Education Journal

EISSN: 1923-1202
Publisher: University of Saskatchewan

ARTICLE INFORMATION
==============================
Subjects: Dp 11–7

Realignment of an Established Faculty Development Program for New teachers: A Systematic Approach

Antao Viola University of Toronto

Lazor Jana University of Toronto

Bordman Risa University of Toronto

Colledge Eleanor University of Toronto

Crispino Natascha University of Toronto

Gabor Rob University of Toronto

Goldstein Susan University of Toronto

Branigan Monica University of Toronto

Homer Michelle University of Toronto

Ivanovic Maria University of Toronto

Jain Rahul University of Toronto

Kates Michael University of Toronto

Lemieux Camille University of Toronto

Kopansky-Giles Deborah University of Toronto

Markovski Milena University of Toronto

Roberts Michael University of Toronto

Schram Carrie University of Toronto

Stoller Rebecca University of Toronto

Weisdorf Thea University of Toronto

Zimcik Heather University of Toronto

Penciner Rick University of Toronto

Sampson Gwen University of Toronto

Electronic publication date: 2019 Apr 3

JOURNAL INFORMATION
==============================
NLM Title Abbreviation: Can Med Educ J
Journal ID: Can Med Educ J
NLM Journal ID: 101560935
Journal ID: CMEJ

Canadian Medical Education Journal

EISSN: 1923-1202
Publisher: University of Saskatchewan

ARTICLE INFORMATION
==============================
Subjects: Dp 11–8

Just what I wanted to hear: Target teaching tips by podcast

Jalloh Chelsea University of Manitoba

Yurkiw Steve University of Manitoba

Ens Anita University of Manitoba

Electronic publication date: 2019 Apr 3

JOURNAL INFORMATION
==============================
NLM Title Abbreviation: Can Med Educ J
Journal ID: Can Med Educ J
NLM Journal ID: 101560935
Journal ID: CMEJ

Canadian Medical Education Journal

EISSN: 1923-1202
Publisher: University of Saskatchewan

ARTICLE INFORMATION
==============================
Subjects: Dp 12–1

The Development and Implementation of a Novel Simulation-based Interprofessional Trauma Education Module for Pre-licensure Health Professions Students

Patrick Lucy Dalhousie University

Beatty Lorri Dalhousie University

Miller Stephen Dalhousie University

Lackie Kelly Dalhousie University

Electronic publication date: 2019 Apr 3

JOURNAL INFORMATION
==============================
NLM Title Abbreviation: Can Med Educ J
Journal ID: Can Med Educ J
NLM Journal ID: 101560935
Journal ID: CMEJ

Canadian Medical Education Journal

EISSN: 1923-1202
Publisher: University of Saskatchewan

ARTICLE INFORMATION
==============================
Subjects: Dp 12–2

Dispensing Distributed Education: Interprofessional pharmacology training of McMaster Physician Assistant Students by Waterloo School of Pharmacy Students

Burrows Kristen McMaster University

Bowles-Jordan Janie Ms.

Electronic publication date: 2019 Apr 3

JOURNAL INFORMATION
==============================
NLM Title Abbreviation: Can Med Educ J
Journal ID: Can Med Educ J
NLM Journal ID: 101560935
Journal ID: CMEJ

Canadian Medical Education Journal

EISSN: 1923-1202
Publisher: University of Saskatchewan

ARTICLE INFORMATION
==============================
Subjects: Dp 12–6

When 'mental health' meets 'interprofessionalism' meets 'social innovation': A disruptive curriculum for disruptive impact

MacDougall Arlene Dr. Western University

Sule Raksha University of Toronto

Ruhara Ruth Africa Mental Health Foundation

Rodger Susan Dr. Western University

Mutiso Victoria Dr. Africa Mental Health Foundation

Ndetei David Western University

Wathen Nadine Dr. Western University

Janzen Le Ber Marlene Dr. Western University

Branzei Oana Dr. Western University

Njenga Michael Users and Survivors of Psychiatry - Kenya

Saxton Kaitlin Western University

Electronic publication date: 2019 Apr 3

JOURNAL INFORMATION
==============================
NLM Title Abbreviation: Can Med Educ J
Journal ID: Can Med Educ J
NLM Journal ID: 101560935
Journal ID: CMEJ

Canadian Medical Education Journal

EISSN: 1923-1202
Publisher: University of Saskatchewan

ARTICLE INFORMATION
==============================
Subjects: Dp 13–2

Transition to Practice: Evaluating the need for formal training in supervision and assessment among senior emergency medicine residents and new to practice emergency physicians

Kilbertus Sarah University of Toronto

Pardhan Kaif University of Toronto

Zaheer Juveria University of Toronto

Bandiera Glen University of Toronto

Electronic publication date: 2019 Apr 3

JOURNAL INFORMATION
==============================
NLM Title Abbreviation: Can Med Educ J
Journal ID: Can Med Educ J
NLM Journal ID: 101560935
Journal ID: CMEJ

Canadian Medical Education Journal

EISSN: 1923-1202
Publisher: University of Saskatchewan

ARTICLE INFORMATION
==============================
Subjects: Dp 13–3

Developing role models in clinical settings

Ijaz Haider Sonia The Aga Khan University

Gill Roger The Aga Khan University

Riaz Qamar The Aga Khan University

Electronic publication date: 2019 Apr 3

JOURNAL INFORMATION
==============================
NLM Title Abbreviation: Can Med Educ J
Journal ID: Can Med Educ J
NLM Journal ID: 101560935
Journal ID: CMEJ

Canadian Medical Education Journal

EISSN: 1923-1202
Publisher: University of Saskatchewan

ARTICLE INFORMATION
==============================
Subjects: Dp 13–4

Palliative and End-of-Life Care Programs: Preceptorships, Mentoring, and Case-Based Learning to Build Capacity

Harle Ingrid Queen’s University

Smith Karen Queen’s University

McDiarmid Laura Queen’s University

Soleas Eleftherios Queen’s University

Evans Katie Queen’s University

Fraser Bryn Queen’s University

Electronic publication date: 2019 Apr 3

JOURNAL INFORMATION
==============================
NLM Title Abbreviation: Can Med Educ J
Journal ID: Can Med Educ J
NLM Journal ID: 101560935
Journal ID: CMEJ

Canadian Medical Education Journal

EISSN: 1923-1202
Publisher: University of Saskatchewan

ARTICLE INFORMATION
==============================
Subjects: Dp 13–5

ARLO: The role of feedback in a CPD environment

Rezmovitz Jeremy University of Toronto

Wooster Elizabeth OISE/University of Toronto

Maniate Jerry University of Ottawa

MacPhee Ian University of Toronto

Electronic publication date: 2019 Apr 3

JOURNAL INFORMATION
==============================
NLM Title Abbreviation: Can Med Educ J
Journal ID: Can Med Educ J
NLM Journal ID: 101560935
Journal ID: CMEJ

Canadian Medical Education Journal

EISSN: 1923-1202
Publisher: University of Saskatchewan

ARTICLE INFORMATION
==============================
Subjects: Dp 13–7

Lessons Learned: A multi methods systems approach to improving the culture and practice of feedback

Glover Takahashi Susan University of Toronto

Dube Rebecca University of Toronto

Electronic publication date: 2019 Apr 3

JOURNAL INFORMATION
==============================
NLM Title Abbreviation: Can Med Educ J
Journal ID: Can Med Educ J
NLM Journal ID: 101560935
Journal ID: CMEJ

Canadian Medical Education Journal

EISSN: 1923-1202
Publisher: University of Saskatchewan

ARTICLE INFORMATION
==============================
Subjects: Dp 14–2

Revamping senior resident Scenario Rounds using curriculum mapping and a constructivist approach

Venus Kevin University of Toronto

Melvin Lindsay University of Toronto

Shah Rupal University of Toronto

Electronic publication date: 2019 Apr 3

JOURNAL INFORMATION
==============================
NLM Title Abbreviation: Can Med Educ J
Journal ID: Can Med Educ J
NLM Journal ID: 101560935
Journal ID: CMEJ

Canadian Medical Education Journal

EISSN: 1923-1202
Publisher: University of Saskatchewan

ARTICLE INFORMATION
==============================
Subjects: Dp 14–3

25 Years of Distributed Medical Education at a Rural Manitoba Community

Francois Jose University of Manitoba

Kish Scott University of Manitoba

Klassen Don University of Manitoba

Martin Bruce University of Manitoba

Grant Karent University of Manitoba

Electronic publication date: 2019 Apr 3

JOURNAL INFORMATION
==============================
NLM Title Abbreviation: Can Med Educ J
Journal ID: Can Med Educ J
NLM Journal ID: 101560935
Journal ID: CMEJ

Canadian Medical Education Journal

EISSN: 1923-1202
Publisher: University of Saskatchewan

ARTICLE INFORMATION
==============================
Subjects: Dp 14–4

If you build it they will come and stay: Implementing a new Family Medicine Residency Program (FMRP) in an under-served community

Murdoch Stuart University of Toronto

Whittaker Mary-Kay University of Toronto

Rozmovits Linda University of Toronto

Abrahams Caroline University of Toronto

Freeman Risa University of Toronto

Electronic publication date: 2019 Apr 3

JOURNAL INFORMATION
==============================
NLM Title Abbreviation: Can Med Educ J
Journal ID: Can Med Educ J
NLM Journal ID: 101560935
Journal ID: CMEJ

Canadian Medical Education Journal

EISSN: 1923-1202
Publisher: University of Saskatchewan

ARTICLE INFORMATION
==============================
Subjects: Dp 14–5

Identifying the supports and barriers to the implementation of an educational intervention to promote residents' use of Evidence-Based Medicine: a knowledge translation study

Cassis Chantal McGill

Thomas Aliki McGill

Al Zoubi Fadi McGill

Owens Heather McGill

Electronic publication date: 2019 Apr 3

JOURNAL INFORMATION
==============================
NLM Title Abbreviation: Can Med Educ J
Journal ID: Can Med Educ J
NLM Journal ID: 101560935
Journal ID: CMEJ

Canadian Medical Education Journal

EISSN: 1923-1202
Publisher: University of Saskatchewan

ARTICLE INFORMATION
==============================
Subjects: Dp 14–7

Designing innovative programs in Translational Medicine: A case of Developmental Evaluation

Yan Wei Queen’s University

Yan Lam Chi Queen’s University

Archer Stephen Queen’s University

Vanner Stephen Queen’s University

Taylor David Queen’s University

James Paula Queen’s University

Electronic publication date: 2019 Apr 3

JOURNAL INFORMATION
==============================
NLM Title Abbreviation: Can Med Educ J
Journal ID: Can Med Educ J
NLM Journal ID: 101560935
Journal ID: CMEJ

Canadian Medical Education Journal

EISSN: 1923-1202
Publisher: University of Saskatchewan

ARTICLE INFORMATION
==============================
Subjects: Dp 14–8

Post-graduate training in autism-spectrum disorder (ASD) and intellectual disabilities (ID): a review

Adirim Zachary University of Toronto

Thakur Anupam University of Toronto

Electronic publication date: 2019 Apr 3

JOURNAL INFORMATION
==============================
NLM Title Abbreviation: Can Med Educ J
Journal ID: Can Med Educ J
NLM Journal ID: 101560935
Journal ID: CMEJ

Canadian Medical Education Journal

EISSN: 1923-1202
Publisher: University of Saskatchewan

ARTICLE INFORMATION
==============================
Subjects: Dp 15–2

University of Alberta Professionalism: A Transparent, Supportive, & Consistent Approach to Professionalism Concern Reporting, Process & Intervention

Smyth Penelope University of Alberta

Fisher Bruce University of Alberta

Ganatra Seema University of Alberta

Gowrishankar Manjula University of Alberta

Jones Britney University of Alberta

Ma Cary University of Alberta

Persad Sujata University of Alberta

Walton Jennifer University of Alberta

Widder Sandy University of Alberta

Dennis Kunimoto University of Alberta

Electronic publication date: 2019 Apr 3

JOURNAL INFORMATION
==============================
NLM Title Abbreviation: Can Med Educ J
Journal ID: Can Med Educ J
NLM Journal ID: 101560935
Journal ID: CMEJ

Canadian Medical Education Journal

EISSN: 1923-1202
Publisher: University of Saskatchewan

ARTICLE INFORMATION
==============================
Subjects: Dp 15–3

Knowledge and implementation of current opioid guidelines among health care providers

C Sanchez Ramirez Diana University of Manitoba

Polimeni Christine University of Manitoba

Electronic publication date: 2019 Apr 3

JOURNAL INFORMATION
==============================
NLM Title Abbreviation: Can Med Educ J
Journal ID: Can Med Educ J
NLM Journal ID: 101560935
Journal ID: CMEJ

Canadian Medical Education Journal

EISSN: 1923-1202
Publisher: University of Saskatchewan

ARTICLE INFORMATION
==============================
Subjects: Dp 15–4

Adjusting to Duty Hour Reforms: Residents' Perception of the Patient Safety Climate in Interdisciplinary Night-Float Rotations

Harvey Adrien Université Laval

Lafleur Alexandre Université Laval

Simard Caroline Université Laval

Electronic publication date: 2019 Apr 3

JOURNAL INFORMATION
==============================
NLM Title Abbreviation: Can Med Educ J
Journal ID: Can Med Educ J
NLM Journal ID: 101560935
Journal ID: CMEJ

Canadian Medical Education Journal

EISSN: 1923-1202
Publisher: University of Saskatchewan

ARTICLE INFORMATION
==============================
Subjects: Dp 15–6

Changing the World One Toy at a Time

Maruyama Michiko University of Alberta

Lederer Robert University of Alberta

Electronic publication date: 2019 Apr 3

JOURNAL INFORMATION
==============================
NLM Title Abbreviation: Can Med Educ J
Journal ID: Can Med Educ J
NLM Journal ID: 101560935
Journal ID: CMEJ

Canadian Medical Education Journal

EISSN: 1923-1202
Publisher: University of Saskatchewan

ARTICLE INFORMATION
==============================
Subjects: Dp 16–1

Supports and constraints for memorable palliative care learning: implications for emerging professional identity.

Kilbertus Frances Northern Ontario School of Medicine

Ajjawi Rola Deakin University

Electronic publication date: 2019 Apr 3

JOURNAL INFORMATION
==============================
NLM Title Abbreviation: Can Med Educ J
Journal ID: Can Med Educ J
NLM Journal ID: 101560935
Journal ID: CMEJ

Canadian Medical Education Journal

EISSN: 1923-1202
Publisher: University of Saskatchewan

ARTICLE INFORMATION
==============================
Subjects: Dp 16–2

Bringing the voice of primary care to BC Cancer: Results from a province-wide primary care oncology needs assessment

Beamish Laura University of British Columbia

Lynn Brenna University of British Columbia

Clelland Cathy BC Cancer

Born Dawson University of British Columbia

Wolfe Jennifer BC Cancer

Little Kelly Vancouver Division of Family Pracitce

Electronic publication date: 2019 Apr 3

JOURNAL INFORMATION
==============================
NLM Title Abbreviation: Can Med Educ J
Journal ID: Can Med Educ J
NLM Journal ID: 101560935
Journal ID: CMEJ

Canadian Medical Education Journal

EISSN: 1923-1202
Publisher: University of Saskatchewan

ARTICLE INFORMATION
==============================
Subjects: Dp 16–4

Exploring the Meaning and Implications of Emotional Safety in Medical Education Using a Peer Mentoring Initiative

Tsuei Sian Department of Global Health and Populations, Harvard T H Chan School of Public Health, Harvard University; School of Population and Public Health, UBC

Lee Dongho University of British Columbia

Ho Charles University of British Columbia

Regehr Glenn University of British Columbia

Nimmon Laura University of British Columbia

Electronic publication date: 2019 Apr 3

JOURNAL INFORMATION
==============================
NLM Title Abbreviation: Can Med Educ J
Journal ID: Can Med Educ J
NLM Journal ID: 101560935
Journal ID: CMEJ

Canadian Medical Education Journal

EISSN: 1923-1202
Publisher: University of Saskatchewan

ARTICLE INFORMATION
==============================
Subjects: Dp 16–5

Providing Education, Improving Access: Training Nurse Practitioners (NPs) to Provide HIV Treatment and Prevention in British Columbia (BC) - A Novel Interdisciplinary HIV Training Program

Puskas Cathy British Columbia Centre for Excellence in HIV/AIDS

Guillemi Silvia British Columbia Centre for Excellence in HIV/AIDS

Koleszar Karah British Columbia Centre for Excellence in HIV/AIDS

Beaveridge Jennifer Vancouver Coastal Health Authority

Nicholson Donna Fraser Health Authority

Payne Martin Dr. Peter AIDS Foundation

Electronic publication date: 2019 Apr 3

JOURNAL INFORMATION
==============================
NLM Title Abbreviation: Can Med Educ J
Journal ID: Can Med Educ J
NLM Journal ID: 101560935
Journal ID: CMEJ

Canadian Medical Education Journal

EISSN: 1923-1202
Publisher: University of Saskatchewan

ARTICLE INFORMATION
==============================
Subjects: Dp 16–6

A Description of the Professional Identity of Massage Therapists in Ontario

Baskwill Amanda McMaster University

Dore Kelly McMaster University

Vanstone Meredith McMaster University

Harnish Del McMaster University

Electronic publication date: 2019 Apr 3

JOURNAL INFORMATION
==============================
NLM Title Abbreviation: Can Med Educ J
Journal ID: Can Med Educ J
NLM Journal ID: 101560935
Journal ID: CMEJ

Canadian Medical Education Journal

EISSN: 1923-1202
Publisher: University of Saskatchewan

ARTICLE INFORMATION
==============================
Subjects: Dp 16–8

The transition of International Medical Graduate (IMG) fellows into the ICU team: Identity Gain or Loss?

Piquette Dominique University of Toronto

Mecklenburg Anne University of Toronto

Najeeb Umberin University of Toronto

Rose Louise University of Toronto

Lingard Lorelei Western University,

Electronic publication date: 2019 Apr 3

JOURNAL INFORMATION
==============================
NLM Title Abbreviation: Can Med Educ J
Journal ID: Can Med Educ J
NLM Journal ID: 101560935
Journal ID: CMEJ

Canadian Medical Education Journal

EISSN: 1923-1202
Publisher: University of Saskatchewan

ARTICLE INFORMATION
==============================
Subjects: Dp 17–1

Using Mixed Methods and Multiple Data Sources to Assess Comparability of Sites in Clerkship Rotations

Mawdsley Helen University of Manitoba

Electronic publication date: 2019 Apr 3

JOURNAL INFORMATION
==============================
NLM Title Abbreviation: Can Med Educ J
Journal ID: Can Med Educ J
NLM Journal ID: 101560935
Journal ID: CMEJ

Canadian Medical Education Journal

EISSN: 1923-1202
Publisher: University of Saskatchewan

ARTICLE INFORMATION
==============================
Subjects: Dp 17–4

Halving your Paperwork: The Administrative and Emotional Benefits of Combined CPD and FD forms

Kittner Kate Queen’s University

Soleas Eleftherios Queen’s University

James Allyson Queen’s University

De Sousa Mikaila Queen’s University

Scott Lee Carolyn Queen’s University

Electronic publication date: 2019 Apr 3

JOURNAL INFORMATION
==============================
NLM Title Abbreviation: Can Med Educ J
Journal ID: Can Med Educ J
NLM Journal ID: 101560935
Journal ID: CMEJ

Canadian Medical Education Journal

EISSN: 1923-1202
Publisher: University of Saskatchewan

ARTICLE INFORMATION
==============================
Subjects: Dp 17–5

Resident Engagement in Accreditation

Colbourne Terry University of Manitoba

Krawchenko-Shawarsky Adriana University of Manitoba

Maniate Jerry University of Ottawa

Moores Margaret University of Ottawa

Mungroo Rani Resident Doctors of Canada

Electronic publication date: 2019 Apr 3

JOURNAL INFORMATION
==============================
NLM Title Abbreviation: Can Med Educ J
Journal ID: Can Med Educ J
NLM Journal ID: 101560935
Journal ID: CMEJ

Canadian Medical Education Journal

EISSN: 1923-1202
Publisher: University of Saskatchewan

ARTICLE INFORMATION
==============================
Subjects: Dp 17–6

Graduate studies for Clinician Educators: a continuum of learning perspective at Laval University

Saucier Danielle Université Laval

Gingras Nathalie Université Laval

Beaulieu Andréane Université Laval

Electronic publication date: 2019 Apr 3

JOURNAL INFORMATION
==============================
NLM Title Abbreviation: Can Med Educ J
Journal ID: Can Med Educ J
NLM Journal ID: 101560935
Journal ID: CMEJ

Canadian Medical Education Journal

EISSN: 1923-1202
Publisher: University of Saskatchewan

ARTICLE INFORMATION
==============================
Subjects: Dp 17–7

Conflict of interest numbers after establishing reporting mechanisms in clerkship

Veale Pamela University of Calgary

Paget Mike University of Calgary

Electronic publication date: 2019 Apr 3

JOURNAL INFORMATION
==============================
NLM Title Abbreviation: Can Med Educ J
Journal ID: Can Med Educ J
NLM Journal ID: 101560935
Journal ID: CMEJ

Canadian Medical Education Journal

EISSN: 1923-1202
Publisher: University of Saskatchewan

ARTICLE INFORMATION
==============================
Subjects: Dp 18–1

A New Technique for Considering Point of View in History of Medicine Research

Nash Carol University of Toronto

Electronic publication date: 2019 Apr 3

JOURNAL INFORMATION
==============================
NLM Title Abbreviation: Can Med Educ J
Journal ID: Can Med Educ J
NLM Journal ID: 101560935
Journal ID: CMEJ

Canadian Medical Education Journal

EISSN: 1923-1202
Publisher: University of Saskatchewan

ARTICLE INFORMATION
==============================
Subjects: Dp 18–2

Was it fair? Evaluating predictors of successful funding to an education scholarship grant

Farrugia Michele University of Toronto

Kulasegraram Mahan University of Toronto

Cartmill Carrie University of Toronto

Fechtig Lindsay University of Toronto

Khan Tasnia University of Toronto

Paton Morag University of Toronto

Freeman Risa University of Toronto

Electronic publication date: 2019 Apr 3

JOURNAL INFORMATION
==============================
NLM Title Abbreviation: Can Med Educ J
Journal ID: Can Med Educ J
NLM Journal ID: 101560935
Journal ID: CMEJ

Canadian Medical Education Journal

EISSN: 1923-1202
Publisher: University of Saskatchewan

ARTICLE INFORMATION
==============================
Subjects: Dp 18–3

A Critical Review of Representation in Global Oncology Curricula Development and the Influence of Neocolonialism

Giuliani Meredith University of Toronto

Frambach Janneke Maastricht University

Broadhurst Michaela Princess Margaret Cancer Centre

Fazelzad Rouhi Princess Margaret Cancer Centre

Papadakos Janet Princess Margaret Cancer Centre

Driessen Erik Maastricht University

Athina (Tina) Martimianakis Maria University of Toronto

Electronic publication date: 2019 Apr 3

JOURNAL INFORMATION
==============================
NLM Title Abbreviation: Can Med Educ J
Journal ID: Can Med Educ J
NLM Journal ID: 101560935
Journal ID: CMEJ

Canadian Medical Education Journal

EISSN: 1923-1202
Publisher: University of Saskatchewan

ARTICLE INFORMATION
==============================
Subjects: Dp 18–4

Barriers and Facilitators to Research by Schulich School of Medicine & Dentistry Physicians Located in Distributed Sites

Doan Lynn Western University

Romagnoli Tommaso Western University

Taylor Madeline Western University

Kim Danny Western University

Kim George Western University

Electronic publication date: 2019 Apr 3

JOURNAL INFORMATION
==============================
NLM Title Abbreviation: Can Med Educ J
Journal ID: Can Med Educ J
NLM Journal ID: 101560935
Journal ID: CMEJ

Canadian Medical Education Journal

EISSN: 1923-1202
Publisher: University of Saskatchewan

ARTICLE INFORMATION
==============================
Subjects: Dp 18–5

Investigating medical student perceptions of the influence of health science research training on their learning experiences and approach to patient care: A program evaluation study

Smith-Gorvie Telisha University of Toronto

Nyhof-Young Joyce University of Toronto

D'Urzo Tony University of Toronto

Katzman Debra University of Toronto

Electronic publication date: 2019 Apr 3

JOURNAL INFORMATION
==============================
NLM Title Abbreviation: Can Med Educ J
Journal ID: Can Med Educ J
NLM Journal ID: 101560935
Journal ID: CMEJ

Canadian Medical Education Journal

EISSN: 1923-1202
Publisher: University of Saskatchewan

ARTICLE INFORMATION
==============================
Subjects: Dp 18–7

Evaluation of the effectiveness of a hybrid-course model in the delivery of an introductory program in clinical epidemiology and biostatistics for healthcare professionals

Famure Olusegun University of Toronto

Minkovich Michelle University of Toronto

Clotea Ioana University of Toronto

Li George University of Toronto

Kim Joseph University of Toronto

Famure Olusegun University of Toronto

Electronic publication date: 2019 Apr 3

JOURNAL INFORMATION
==============================
NLM Title Abbreviation: Can Med Educ J
Journal ID: Can Med Educ J
NLM Journal ID: 101560935
Journal ID: CMEJ

Canadian Medical Education Journal

EISSN: 1923-1202
Publisher: University of Saskatchewan

ARTICLE INFORMATION
==============================
Subjects: Dp 18–8

Seeing the Patient: The Use of Portraiture and Narrative to Explore the Lived Experience of Children with Epilepsy and their Caregivers

Stewart Wendy Dalhousie University

Gilbert Mark Dalhousie University

MacGillivray Melanie Dalhousie University

Electronic publication date: 2019 Apr 3

JOURNAL INFORMATION
==============================
NLM Title Abbreviation: Can Med Educ J
Journal ID: Can Med Educ J
NLM Journal ID: 101560935
Journal ID: CMEJ

Canadian Medical Education Journal

EISSN: 1923-1202
Publisher: University of Saskatchewan

ARTICLE INFORMATION
==============================
Subjects: Dp 19–1

Using Self-Reported Measures of Confidence and Anxiety to Determine the Efficacy of the Surgical Exploration And Discovery (SEAD) Program in Reducing Anxiety and Increasing Confidence in Performing Procedural Skills

Battaglia Frank University of Ottawa

Langlois Emilie University of Ottawa

Market Marisa University of Ottawa

Shin John University of Ottawa

Seabrook Christine University of Ottawa

Brandys Tim University of Ottawa

Electronic publication date: 2019 Apr 3

JOURNAL INFORMATION
==============================
NLM Title Abbreviation: Can Med Educ J
Journal ID: Can Med Educ J
NLM Journal ID: 101560935
Journal ID: CMEJ

Canadian Medical Education Journal

EISSN: 1923-1202
Publisher: University of Saskatchewan

ARTICLE INFORMATION
==============================
Subjects: Dp 19–2

Office Emergencies: A Novel Simulation-Based CPD Workshop

Waldolf Richard University of Ottawa

Waldolf Richard University of Ottawa

Éthier Estelle La Cité Collégiale

Parent Nicole Association des médecins francophones du Canada

Michon Alain Institut du Savoir Montfort

Montgomery George University of Ottawa

Electronic publication date: 2019 Apr 3

JOURNAL INFORMATION
==============================
NLM Title Abbreviation: Can Med Educ J
Journal ID: Can Med Educ J
NLM Journal ID: 101560935
Journal ID: CMEJ

Canadian Medical Education Journal

EISSN: 1923-1202
Publisher: University of Saskatchewan

ARTICLE INFORMATION
==============================
Subjects: Dp 19–4

Outside the comfort zone: Evaluation of a simulation-based curriculum in managing agitated patients for paediatric residents

Fleming Lindsay University of Toronto

Hick Katherine University of Toronto

Kulkarni Chetana University of Toronto

Lorber Sharon University of Toronto

Electronic publication date: 2019 Apr 3

JOURNAL INFORMATION
==============================
NLM Title Abbreviation: Can Med Educ J
Journal ID: Can Med Educ J
NLM Journal ID: 101560935
Journal ID: CMEJ

Canadian Medical Education Journal

EISSN: 1923-1202
Publisher: University of Saskatchewan

ARTICLE INFORMATION
==============================
Subjects: Dp 19–5

Creation and Evaluation of a Standardized Sustainable Curriculum To Teach Perineal Laceration Repair To Maternity Care Providers

Caccia Nicolette University of Toronto

Kline Kathleen Ryerson University

Kiriloff Elena Ryerson University

Evans Victoria Ryerson University

Van Wagner Vicki Ryerson University

Lynch Brigit McMaster University

Electronic publication date: 2019 Apr 3

JOURNAL INFORMATION
==============================
NLM Title Abbreviation: Can Med Educ J
Journal ID: Can Med Educ J
NLM Journal ID: 101560935
Journal ID: CMEJ

Canadian Medical Education Journal

EISSN: 1923-1202
Publisher: University of Saskatchewan

ARTICLE INFORMATION
==============================
Subjects: Dp 20–2

Performance Assessment Using Prosections In Anatomy Laboratory Teaching

Leo Joyce Royal Jubilee Hospital

Kuo Kuo-Hsing University of British Columbia

Electronic publication date: 2019 Apr 3

JOURNAL INFORMATION
==============================
NLM Title Abbreviation: Can Med Educ J
Journal ID: Can Med Educ J
NLM Journal ID: 101560935
Journal ID: CMEJ

Canadian Medical Education Journal

EISSN: 1923-1202
Publisher: University of Saskatchewan

ARTICLE INFORMATION
==============================
Subjects: Dp 20–5

Case-based learning in undergraduate dermatology medical education

Zhou Linda University of British Columbia

Liu Annie University of Toronto

Lam Andrea University of Toronto

Dahlke Erin University of Toronto

Electronic publication date: 2019 Apr 3

JOURNAL INFORMATION
==============================
NLM Title Abbreviation: Can Med Educ J
Journal ID: Can Med Educ J
NLM Journal ID: 101560935
Journal ID: CMEJ

Canadian Medical Education Journal

EISSN: 1923-1202
Publisher: University of Saskatchewan

ARTICLE INFORMATION
==============================
Subjects: Dp 20–7

Is it time to get serious about play? A Needs Assessment for Medical Improv in the UME Curriculum

Tanwani Jaya University of Toronto

Rezmovitz Jeremy University of Toronto

Peranson Judith University of Toronto

Electronic publication date: 2019 Apr 3

JOURNAL INFORMATION
==============================
NLM Title Abbreviation: Can Med Educ J
Journal ID: Can Med Educ J
NLM Journal ID: 101560935
Journal ID: CMEJ

Canadian Medical Education Journal

EISSN: 1923-1202
Publisher: University of Saskatchewan

ARTICLE INFORMATION
==============================
Subjects: Dp 20–8

Multi-modal education approach to enhance the learning of a difficult airway pathway for non-anesthetists in non-operating room settings: The Niagara Health Experience

Shira Brown N. Niagara Health

Chirico John Niagara Health

Hollidge Melanie Niagara Health

Caetano Helen Niagara Health

Electronic publication date: 2019 Apr 3

JOURNAL INFORMATION
==============================
NLM Title Abbreviation: Can Med Educ J
Journal ID: Can Med Educ J
NLM Journal ID: 101560935
Journal ID: CMEJ

Canadian Medical Education Journal

EISSN: 1923-1202
Publisher: University of Saskatchewan

ARTICLE INFORMATION
==============================
Subjects: Dp 21–1

Virtual Pathology: An effective teaching tool for Medical Students

Prasad Chaya Western University Of Health Sciences

Electronic publication date: 2019 Apr 3

JOURNAL INFORMATION
==============================
NLM Title Abbreviation: Can Med Educ J
Journal ID: Can Med Educ J
NLM Journal ID: 101560935
Journal ID: CMEJ

Canadian Medical Education Journal

EISSN: 1923-1202
Publisher: University of Saskatchewan

ARTICLE INFORMATION
==============================
Subjects: Dp 21–2

Clinical Informatics Competencies Betwixt Canada and the United States: Lessons from a Comparison of Enabling and Sub-Competencies

Chartash David Yale University

Chen Elizabeth Brown University

Rosenman Marc Northwestern University

Electronic publication date: 2019 Apr 3

JOURNAL INFORMATION
==============================
NLM Title Abbreviation: Can Med Educ J
Journal ID: Can Med Educ J
NLM Journal ID: 101560935
Journal ID: CMEJ

Canadian Medical Education Journal

EISSN: 1923-1202
Publisher: University of Saskatchewan

ARTICLE INFORMATION
==============================
Subjects: Dp 21–3

One hundred thousand hours: data from a decade on YouTube

Topps David University of Calgary

Ellaway Rachel University of Calgary

Electronic publication date: 2019 Apr 3

JOURNAL INFORMATION
==============================
NLM Title Abbreviation: Can Med Educ J
Journal ID: Can Med Educ J
NLM Journal ID: 101560935
Journal ID: CMEJ

Canadian Medical Education Journal

EISSN: 1923-1202
Publisher: University of Saskatchewan

ARTICLE INFORMATION
==============================
Subjects: Dp 21–4

Smartphone use among medical students at Thammasat medical school: the impact on medical education

Sritipsukho Paskorn Thammasat University

Electronic publication date: 2019 Apr 3

JOURNAL INFORMATION
==============================
NLM Title Abbreviation: Can Med Educ J
Journal ID: Can Med Educ J
NLM Journal ID: 101560935
Journal ID: CMEJ

Canadian Medical Education Journal

EISSN: 1923-1202
Publisher: University of Saskatchewan

ARTICLE INFORMATION
==============================
Subjects: Dp 21–5

Using Digital Storytelling to Understand the Teaching and Learning of Compassionate Care

Banerjee Debi University of Toronto

Grundland Batya University of Toronto

Murphy Claire University of Toronto

Hum Susan University of Toronto

Rezmovitz Jeremy University of Toronto

Electronic publication date: 2019 Apr 3

JOURNAL INFORMATION
==============================
NLM Title Abbreviation: Can Med Educ J
Journal ID: Can Med Educ J
NLM Journal ID: 101560935
Journal ID: CMEJ

Canadian Medical Education Journal

EISSN: 1923-1202
Publisher: University of Saskatchewan

ARTICLE INFORMATION
==============================
Subjects: Dp 21–8

Virtual interactive cases in rheumatology medical education

Zhou Linda University of British Columbia

Chow Shirley University of Toronto

Electronic publication date: 2019 Apr 3

JOURNAL INFORMATION
==============================
NLM Title Abbreviation: Can Med Educ J
Journal ID: Can Med Educ J
NLM Journal ID: 101560935
Journal ID: CMEJ

Canadian Medical Education Journal

EISSN: 1923-1202
Publisher: University of Saskatchewan

ARTICLE INFORMATION
==============================
Subjects: Dp 22–3

Providing added value to undergraduate medical education programs: Reliability of accreditation decisions

Blouin Danielle CACMS / CACME

Venance Shannon CACMS / CACME

Electronic publication date: 2019 Apr 3

JOURNAL INFORMATION
==============================
NLM Title Abbreviation: Can Med Educ J
Journal ID: Can Med Educ J
NLM Journal ID: 101560935
Journal ID: CMEJ

Canadian Medical Education Journal

EISSN: 1923-1202
Publisher: University of Saskatchewan

ARTICLE INFORMATION
==============================
Subjects: Dp 22–6

The case-based method to teach population health promotion and prevention in undergraduate MD program

Cambron-Goulet Evelyne Université de Sherbrooke

Baron Geneviève Université de Sherbrooke

Lisée Véronique Université de Sherbrooke

Houde Ghislaine Université de Sherbrooke

Gagné Eve-Reine Université de Sherbrooke

Electronic publication date: 2019 Apr 3

JOURNAL INFORMATION
==============================
NLM Title Abbreviation: Can Med Educ J
Journal ID: Can Med Educ J
NLM Journal ID: 101560935
Journal ID: CMEJ

Canadian Medical Education Journal

EISSN: 1923-1202
Publisher: University of Saskatchewan

ARTICLE INFORMATION
==============================
Subjects: Dp 22–8

Comparing medical student and resident perceptions of preparedness for the third year core surgical clerkship rotation: A needs assessment

Wood Melissa University of Saskatchewan

Domes Trustin University of Saskatchewan

Nguyen Ron University of Saskatchewan

Electronic publication date: 2019 Apr 3

JOURNAL INFORMATION
==============================
NLM Title Abbreviation: Can Med Educ J
Journal ID: Can Med Educ J
NLM Journal ID: 101560935
Journal ID: CMEJ

Canadian Medical Education Journal

EISSN: 1923-1202
Publisher: University of Saskatchewan

ARTICLE INFORMATION
==============================
Subjects: Dp 23–1

Wellness education to address burnout in medical students

Bughi Stephanie Rancho Research Institute at Rancho Los Amigos National Rehabilitation Center

Prasad Chaya Western University Of Health Sciences

Fraix Marcel Western University Of Health Sciences

Bughi Stefan Rancho Research Institute at Rancho Los Amigos National Rehabilitation Center

Electronic publication date: 2019 Apr 3

JOURNAL INFORMATION
==============================
NLM Title Abbreviation: Can Med Educ J
Journal ID: Can Med Educ J
NLM Journal ID: 101560935
Journal ID: CMEJ

Canadian Medical Education Journal

EISSN: 1923-1202
Publisher: University of Saskatchewan

ARTICLE INFORMATION
==============================
Subjects: Dp 23–2

Put Your Mask On First Before Assisting Others! A Wellness Retreat for Students of Peer Support Groups

Mélançon Joanie Université Laval

Lafleur Alexandre Université Laval

Vézina Andrée Université Laval

Melançon Joanie Université Laval

Petitclerc Laurence Université Laval

Electronic publication date: 2019 Apr 3

JOURNAL INFORMATION
==============================
NLM Title Abbreviation: Can Med Educ J
Journal ID: Can Med Educ J
NLM Journal ID: 101560935
Journal ID: CMEJ

Canadian Medical Education Journal

EISSN: 1923-1202
Publisher: University of Saskatchewan

ARTICLE INFORMATION
==============================
Subjects: Dp 23–3

Cultivating Medical Students' Well-Being Through a 4-Year Longitudinal Wellness Curriculum

Velez Camila McGill

Gupta Namta McGill

Gendreau Pascale McGill

Electronic publication date: 2019 Apr 3

JOURNAL INFORMATION
==============================
NLM Title Abbreviation: Can Med Educ J
Journal ID: Can Med Educ J
NLM Journal ID: 101560935
Journal ID: CMEJ

Canadian Medical Education Journal

EISSN: 1923-1202
Publisher: University of Saskatchewan

ARTICLE INFORMATION
==============================
Subjects: Dp 23–4

The Canadian Federation of Medical Students National Wellness Program: A Comprehensive National Student-Led Innovation

Do Victor University of Alberta

Smith Stephanie University of Calgary

Annan Henry Dalhousie University

Rizzuti Franco University of Calgary

Electronic publication date: 2019 Apr 3

JOURNAL INFORMATION
==============================
NLM Title Abbreviation: Can Med Educ J
Journal ID: Can Med Educ J
NLM Journal ID: 101560935
Journal ID: CMEJ

Canadian Medical Education Journal

EISSN: 1923-1202
Publisher: University of Saskatchewan

ARTICLE INFORMATION
==============================
Subjects: Dp 23–5

Developing and implementing interactive 360-degree video simulations, a form of immersive reality, to prepare learners for emotionally challenging clinical experiences.

Blank Gabriel University of British Columbia

Anthony Joseph University of British Columbia

Parhar Gurdeep University of British Columbia

Electronic publication date: 2019 Apr 3

JOURNAL INFORMATION
==============================
NLM Title Abbreviation: Can Med Educ J
Journal ID: Can Med Educ J
NLM Journal ID: 101560935
Journal ID: CMEJ

Canadian Medical Education Journal

EISSN: 1923-1202
Publisher: University of Saskatchewan

ARTICLE INFORMATION
==============================
Subjects: Dp 23–6

Physician Well-being as an Entrustable Professional Activity

Gregory Jonathan Western University

Sukhera Javeed Western University

Ali Bateman E. Western University

Electronic publication date: 2019 Apr 3

JOURNAL INFORMATION
==============================
NLM Title Abbreviation: Can Med Educ J
Journal ID: Can Med Educ J
NLM Journal ID: 101560935
Journal ID: CMEJ

Canadian Medical Education Journal

EISSN: 1923-1202
Publisher: University of Saskatchewan

ARTICLE INFORMATION
==============================
Subjects: Dp 23–7

Is Transition to Competency by Design Associated With Faculty Burnout?

Fraser Amy University of Ottawa

Ritchie Kerri The Ottawa Hospital

Sydor Devin Queen’s University

Gerin-Lajoie Caroline University of Ottawa

Electronic publication date: 2019 Apr 3

JOURNAL INFORMATION
==============================
NLM Title Abbreviation: Can Med Educ J
Journal ID: Can Med Educ J
NLM Journal ID: 101560935
Journal ID: CMEJ

Canadian Medical Education Journal

EISSN: 1923-1202
Publisher: University of Saskatchewan

ARTICLE INFORMATION
==============================
Subjects: Dp 23–8

Does Burnout Predict Teacher Self-Efficacy in Academic Physicians?

Fraser Amy University of Ottawa

Ritchie Kerri University of Ottawa

Sydor Devin Queen’s University

Gerin-Lajoie Caroline University of Ottawa

Electronic publication date: 2019 Apr 3

